# Preliminary study of Cell Wall Structure and its Mechanical Properties of C3H and HCT RNAi Transgenic Poplar Sapling

**DOI:** 10.1038/s41598-018-28675-5

**Published:** 2018-07-12

**Authors:** Xianwu Zhou, Suhong Ren, Mengzhu Lu, Shutang Zhao, Zhangjing Chen, Rongjun Zhao, Jianxiong Lv

**Affiliations:** 1 State Key Laboratory of Tree Genetics and Breeding, Research Institute of Wood Industry, Chinese Academy of Forestry, 100091 Beijing, P.R. China; 2 State Key Laboratory of Tree Genetics and Breeding, Research Institute of Forestry, Chinese Academy of Forestry, 100091 Beijing, P.R. China; 3 Department of Wood Science and Forest Products, Virginia Polytechnic Institute and State University, Blacksburg, USA

## Abstract

This research focused on the cell wall structure and its mechanical properties of down-regulated Coumaroyl shikimate 3-hydroxylase (C3H) transgenic poplar and down-regulated hydroxycinnamoyl CoA: shikimate hydroxycinnamoyl transferase (HCT) transgenic poplar (*Populus alba* × *P*. *glandulosa* cv ‘84 k’). The wood samples with respect to microstructure, the longitudinal elastic modulus (MOE) and hardness of wood fiber secondary cell wall were investigated. The results show that the lignin contents in the two transgenic poplar woods were lower than non-modified wood. The C3H transgenic poplar and HCT transgenic poplar have more than 18.5% and 16.1% cellulose crystalline regions than non-modified poplar respectively. The diameter of the fiber cell and the vessel element of transgenic poplars are smaller. Double radial vessel cell wall thicknesses of both transgenic poplars were smaller than non-modified poplar. Cell wall ratios for the transgenic poplar were higher than non-modified poplar and cell wall density was significantly lower in both C3H and HCT transgenic poplar. The cell wall MOEs of C3H and HCT transgenic poplar was 5.8% and 7.0% higher than non-modified poplar. HCT can be more effective than C3H to modify the trees by considerably increasing mechanical properties of the cell wall.

## Introduction

The research of forest transgenic technology started in the early 1980s. In 1986, the transgenic tree *Populus alba* × *grandidentata*^1^ was tried. With the genetic engineering, it is possible to improve wood qualities of the plantation forest. Because of fast growing and vigorous asexual reproduction, poplar is a preferred modified species^2–4^.

The genetic engineering improvement can effectively improve the quality of wood fiber, and thus improve the quality of the pulp^5–11^. The genetic engineering improvement can affect wood Microfibril Angle (MFA)^12,13^. The changes of wood cell morphology and microfibril angle would cause the changes of wood mechanical properties. The wood properties changes with the genetic engineering improvement and many genes have been found to influence the density, shrinkage, swelling and strength of wood.

The genetic engineering technology was used to regulate the biosynthesis process of lignin in trees. This technology can reduce the lignin content or change its structure and composition^14^. There are varieties of enzymes involved in lignin biosynthesis. These enzymes play an important role in lignin synthesis, and the activity of these enzymes is closely related to lignin content^4,15–19^. C4H, 4CL, C3H, CAD, CCR, COMT, CCoAOMT and other genes can reduce significantly lignin content, especially C3H, C4H and 4CL^5–11^. Moreover, lignin is an aromatic compound that is mainly composed of three basic phenylpropane units, guaiacyl unit (G), syringyl unit (S) and *p*-hydroxyphenyl unit (H). The ratio of S and G (S/G) is an important parameter in paper industry^20^, it can be influenced by C3H, COMT, CCoAOMT and F5H^21^. Down-regulation of C3H gene and CCoAOMT gene can increase S/G, which will contribute to delignification^8–11,22^.

Cell morphology of transgenic wood has been studied by the methods of optical microscopy and transmission electron microscopy (TEM)^23^. It has been found that the down-regulated CCR gene can increase the ratio of fiber length to width of transgenic poplar (*Populus* × *euramericana*)^24^. Horvath^25^ studied the effects of lignin content and S/G on the anatomical properties of genetically engineered aspen trees (*Populus tremuloides* Michx.) and found the genetic group with increased S/G ratio had lower diameter growth, lower vessel lumen element diameter, but more numerous vessels. The ratio of cell wall area in cross-section of 4CL transgenic poplar would be slightly different from that of non-modified poplar^26^. Down-regulated CCoAOMT gene can increase fiber width, ratio of cell wall to lumen, cell wall thickness and long fiber (greater than 0.61 mm) distribution frequency and reduce cell lumen diameter^22^. PAD4 gene silencing transgenic poplar had higher cell division rate, it decreased the average cell size but increased the number of cells, decreased the number and diameter of vessel element and increased cell wall thickness^27^.

Since wood properties are largely determined by cell wall structure, and formation of cell wall can be regulated genetically, but research in this area is far from enough. Thus, the objective of this study is to study the cell wall structure and properties of transgenic poplar that can provide some reference for directional cultivation of transgenic poplar. In this study, the effect of transgenic down-regulation Coumaroyl shikimate 3-hydroxylase (C3H) or hydroxycinnamoyl CoA: shikimate hydroxycinnamoyl transferase (HCT) on the wood ultrastructure and mechanical behaviors were studied with the advanced technologies of Glycosyl Linkage Assay, Acetyl Bromide Assay, Fourier transform infrared spectroscopy (FTIR), X-ray diffraction (XRD) and Olympus BX51 optical microscope. Nano indentation (NI) tests were performed to measure the longitudinal elastic modulus and the hardness of wood fiber secondary cell walls.

## Results and Discussion

### Stem Height, Stem Diameter and Internode Number

Table 1 lists the stem height and stem diameter of non-modified poplar and C3H and HCT transgenic poplar. The stem height was measured from the bottom of the stem to the shoot tip and the stem diameter was measured 20 cm above the soil^23^. From the table, there are no difference between heights of non-modified and transgenic species. The stem diameters of transgenic poplars were smaller. The internode numbers of transgenic poplar were less than the non-modified sapling, but there was no significant difference. The down-regulated C3H and HCT genes slowed down the growth of trees in diameter, which is consistent with previous studies^7^.

**Table 1:** Stem height, stem diameter and internode number of non-modified poplar and transgenic poplar.

Sample	Height (cm)	Diameter (mm)	Internode number
Non-modified poplar	141.7 ± 6.7 A	6.05 ± 0.15 A	51 ± 1 A
C3H transgenic poplar	142.7 ± 2.8 A	5.47 ± 0.09 B	49 ± 2 A
HCT transgenic poplar	134.8 ± 6.6 A	5.55 ± 0.33 B	49 ± 3 A
*P-value*	0.178	0.040	0.493

Values after “±” are standard deviations and different letters after values in the same column indicates that there is a significant difference between samples at *p* < 0.05 (Student Newman Keuls test), same in the following tables.

### Cell Morphology

**Figure 1:**
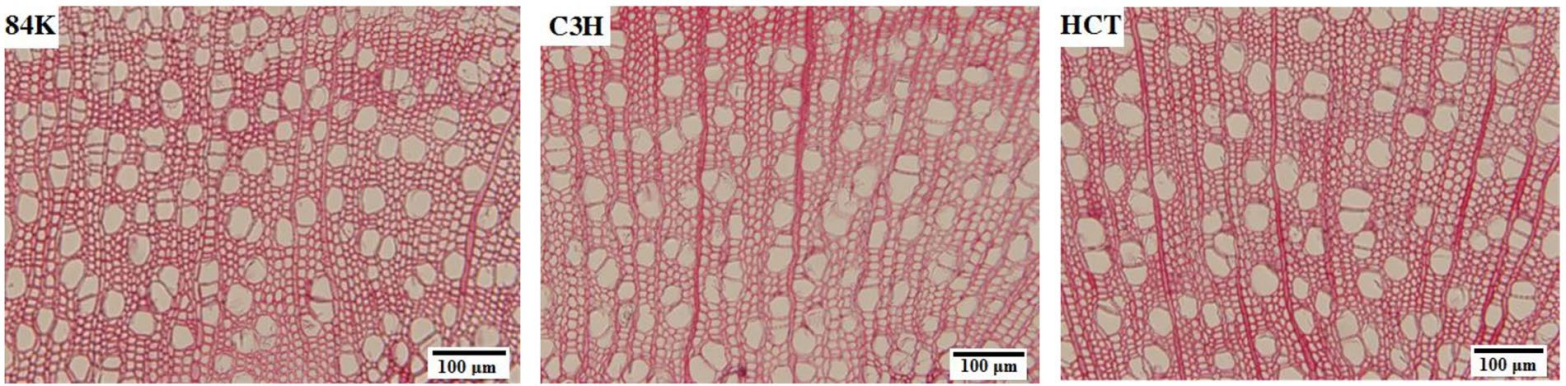
Optical micrograph of non-modified poplar and transgenic poplar.

**Figure 2:**
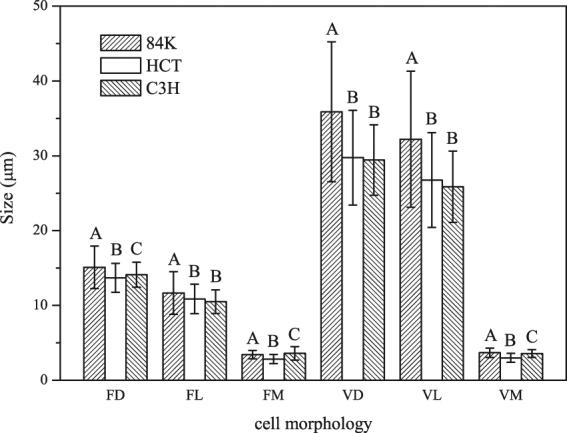
Cell morphology of C3H and HCT RNAi transgenic poplar. FD: Fiber cell diameter, FL: fiber cell lumen diameter, FM: Fiber cell wall thickness, VD: vessel cell diameter, VL: vessel cell lumen diameter, VM: vessel cell wall thickness. The error bars represent standard deviations, and different letters above the columns indicates that there is a significant difference between samples at *p* < 0.05 (Student Newman Keuls test), same in the following figures.

Figure 1 depicts the cross sections of poplar wood with solitary vessel pores and a few of multiple pores. Figure 2 shows the cell morphological changes of C3H and HCT transgenic poplar. Fiber cell diameter and fiber cell lumen diameter of two transgenic poplars are significantly smaller. For the vessels, their cell diameter and cell lumen diameter of both transgenic poplars decreased significantly. Vessel cell wall thickness of HCT transgenic poplar was 18.5% smaller than that of non-modified sapling, and that of C3H transgenic poplar was 3.1% smaller than non-modified.

### Glycosyl Linkage Assay

Table 2 shows the three main cell walls chemical compositional analysis of cell wall from C3H and HCT transgenic poplars. The hemicellulose polymers from C3H samples were 6.6% lower than the control, while that from HCT sample was 8.1% greater than the control. Moreover, the cellulose content of C3H transgenic poplar increased significantly while there was no difference between that of HCT and non-modified poplar. The lignin content of C3H samples and HCT samples were 3.3% and 9.4% lower than non-modified samples respectively. The above results indicate that the reduction of lignin lead to the increase of cellulose in C3H transgenic poplar and the increase of hemicellulose in HCT transgenic poplar.

**Table 2:** Three main cell walls chemical composition analysis of non-modified poplar and transgenic poplar.

Sample	Hemicellulose content (%)	Cellulose content (%)	Lignin content (%)
Non-modified poplar	31.2	43.1	11.1
C3H transgenic poplar	29.2*	47.7*	10.7*
HCT transgenic poplar	33.8*	43.1	10.0*

AIRs sequential extractions were performed as described in Methods. The variance was not shown, but was less than 12%. *Significant difference between the non-modified poplar and transgenic poplar (*t*-test at *p* < 0.05).

### FTIR spectroscopy

Table 3 is the assignment of bands in the FTIR spectrum of cell wall polymers. From Table 4, the characteristic bands ratio I_1507_/I_1425_, I_1507_/I_1382_ and I_1507_/I_895_ of the transgenic poplar show differences from the non-modified poplar. They indicate that the aromatic skeleton of transgenic poplar is significantly lower. This result also confirmed that the lignin content of transgenic poplar is lower after the genetic modification. The increasing bands ratio I_1507_/I_1425_ of transformed poplar revealed that the ratio of aromatic skeleton and C-H in aromatic skeleton rose. The C-H in aromatic skeleton of transgenic poplar fell more sharply than the aromatic skeleton.

**Table 3:** FTIR absorption peak location and assignment.

Wavenumber (cm^−1^)	Absorption Peak Location and Assignment
2900	C-H and CH_2_ stretching
1745	C=O stretching in unconjugated ketone, carbonyl, and aliphatic groups
1596	Aromatic skeletal stretching and C=O stretching (Lignin)
1507	Aromatic skeletal stretching (Lignin)
1462	C-H deformation (Lignin and carbohydrates)
1425	Aromatic skeletal combined with C-H in-plane deforming and stretching (Lignin)
1382	Aliphatic C-H in plane deforming and stretching (Cellulose and hemicellulose)
1332	C-H vibration and C-O vibration in syringyl derivatives
1243	Syringyl ring and C-O stretching (Lignin and xylan)
1158	C-O-C stretching (Cellulose and hemicellulose)
1106	OH association absorption band (cellulose and hemicellulose)
1050	C-O stretching (Cellulose, hemicellulose and lignin)
895	Out of phase ring stretching (Cellulose)

**Table 4:** FTIR relative peak intensity ratio.

Sample	Relative peak intensity ratio				
	I_1507_/I_1425_	I_1507_/I_1382_	I_1507_/I_895_	I_1507_/I_1158_	I_1507_/I_1745_
Non-modified poplar	1.152 ± 0.038 A	1.077 ± 0.056 A	2.770 ± 0.228 A	0.835 ± 0.042 AB	0.373 ± 0.015 A
C3H transgenic poplar	1.399 ± 0.153 B	1.016 ± 0.047 B	2.147 ± 0.147 B	0.809 ± 0.104 A	0.383 ± 0.024 A
HCT transgenic poplar	1.418 ± 0.010 B	1.011 ± 0.065 B	2.154 ± 0.173 B	0.907 ± 0.115 B	0.366 ± 0.038 A
*P-value*	1.44 × 10^−6^	0.024	3.41 × 10^−11^	0.007	0.332

### Measuring relative degree of crystallinity

**Figure 3:**
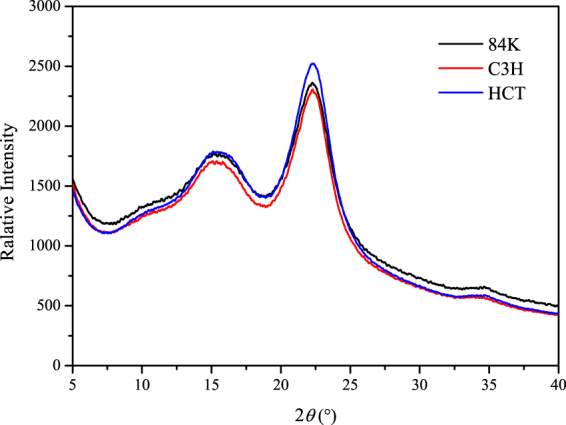
Cellulose crystallinity of non-modified poplar and transgenic poplar.

XRD analysis was used to characterize the crystalline regions of generic modified wood. The crystallinity of non-transformed poplar and transgenic poplar from XRD analysis is shown in Table 5 and Fig. 3. The results show that the cellulose crystallinity of both transgenic poplars were significantly higher than that of non-transformed poplar. The C3H transgenic poplar and HCT transgenic poplar have 18.5% and 16.1% more than non-modified poplar respectively. Li^28^ found that the lower lignin content could increase the cellulose crystallinity. The reduction of lignin may weaken such constraints between hydrogen bonds and surrounding disordered cellulose and lignin and promote tighter packing of crystalline cellulose leading to a greater cellulose crystallinity region^29^.

**Table 5:** Cellulose crystallinity of non-modified poplar and transgenic poplar.

Sample	CrI (%)
Non-modified poplar	34.32 ± 1.74 A
C3H transgenic poplar	40.67 ± 0.27 B
HCT transgenic poplar	39.85 ± 1.05 B
*P-value*	1.77 × 10^−4^

### Acetyl Bromide Assay

**Figure 4:**
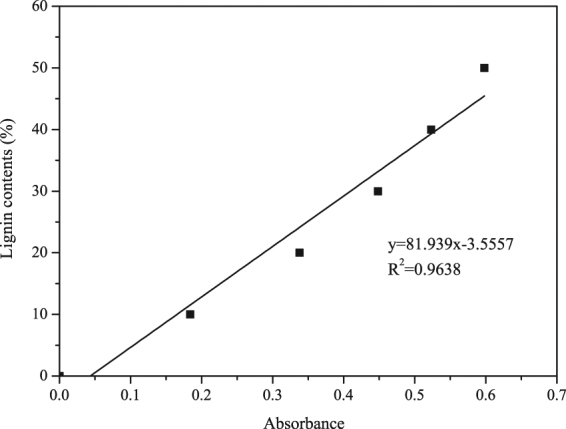
Regression equations obtained from standard curve of alkali lignin content and UV absorbance.

**Figure 5:**
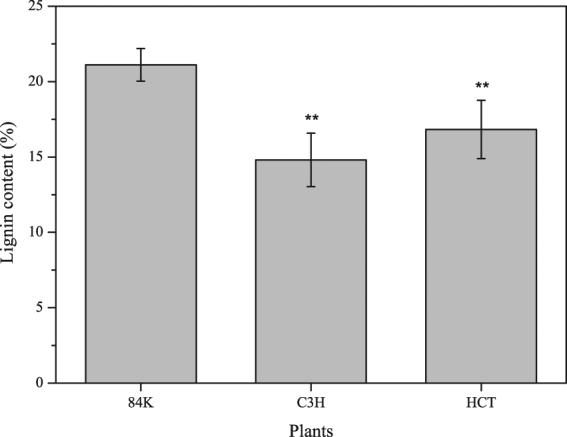
Lignin content of non-modified poplar and transgenic poplar. “**” Represents that there is a significant difference between the non-modified poplar and transgenic poplar at *p* < 0.01 (One-way ANOVA), same in Fig. 6.

Linear regression was performed on the data of lignin content and UV absorbance at 280 nm, and regression equations were obtained from standard curve of alkali lignin, which was y = 81.939 × −3.5557 and R^2^ = 0.9638 (Fig. 4). The lignin content can be obtained by the formula combined with the UV absorbance of the samples as shown in Table 6 and Fig. 5.

**Table 6:** Lignin content of non-modified poplar and transgenic poplar.

Sample	Lignin content (%)
Non-modified poplar	21.11 ± 1.08 A
C3H transgenic poplar	14.80 ± 1.77 B
HCT transgenic poplar	16.82 ± 1.93 C
*P-value*	3.00 × 10^−77^

The results showed that the lignin content of both transgenic poplars lowered markedly, the lignin content of C3H transgenic poplar and HCT gene was decreased by 29.9% and 20.3% respectively. Both Glycosyl Linkage Assay and Acetyl Bromide Assay show that down-regulated C3H and HCT reduced the lignin content of poplar, which is consistent with previous studies^6–10^. Lignin restricts the development of pulp and paper industry result from environmental pollution and the process of bioenergy production from wood^30^. The decrease of lignin content in trees can not only improve economic and environmental benefits in pulp and paper industry, but also contribute to lignocellulose decomposition and improving sugar conversion efficiency^14^. Thus, the down-regulated C3H and HCT transgenic poplars can be better materials in pulp and paper industry and bioenergy production.

### Cell Wall Ratio (CWR) and Basic Density (BD)

**Figure 6:**
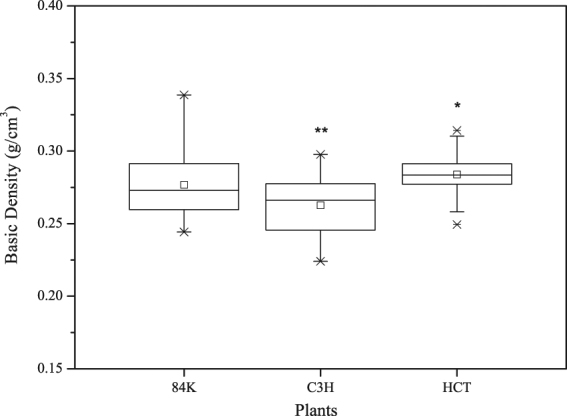
Basic density of non-modified poplar and transgenic poplar. “□” Represents the average value. The line in the box represents the median. The upper and lower edges of the box represent the upper quartile and the lower quartile, respectively. The error bars represent maximum and minimum values, “*”represent that there is a significant difference between the non-modified poplar and transgenic poplar at *p* < 0.05 (One-way ANOVA).

The cell wall ratio is the ratio of cell wall area in wood cross-section, It was significantly higher in both C3H and HCT transgenic poplar because of the lower cell lumen diameter. The basic density of C3H gene and HCT gene down-regulated poplar differed significantly (Table 7 and Fig. 6). The change in C3H transgenic poplar and HCT transgenic poplar basic density was 5.1% and 2.5% less than the non-modified sapling.

**Table 7:** Cell wall ratio and basic density of non-modified poplar and transgenic poplar.

Sample	BD (g/cm^3^)	CWR (%)	Cell wall BD
Non-modified poplar	0.277 ± 0.022 A	40.27 ± 0.54 A	0.688 ± 0.544 A
C3H transgenic poplar	0.263 ± 0.020 B	45.10 ± 1.72 B	0.579 ± 0.496 B
HCT transgenic poplar	0.284 ± 0.012 C	43.71 ± 0.40 B	0.650 ± 0.354 C
*P-value*	3.97 × 10^−11^	2.23 × 10^−5^	1.62 × 10^−3^

Basic density divided by cell wall ratio is cell wall basic density. It decreased significantly in both C3H and HCT transgenic poplar. It indicates that cell wall becomes looser resulting from the decreased lignin content of poplar.

Wood fiber was the major factor to change cell wall ratio. Figure 2 shows that the fiber cell lumen diameter of C3H transgenic poplar decreased, but its fiber cell wall thickness increased, it indicated that there is more substance in C3H transgenic poplar fiber, which can lead to an increase of its cell wall ratio. To the HCT transgenic poplar, both the fiber cell lumen diameter and fiber cell wall thickness decreased, so the reason why its cell wall ratio increased needs further research.

### Cell Wall Mechanical Property

**Figure 7:**
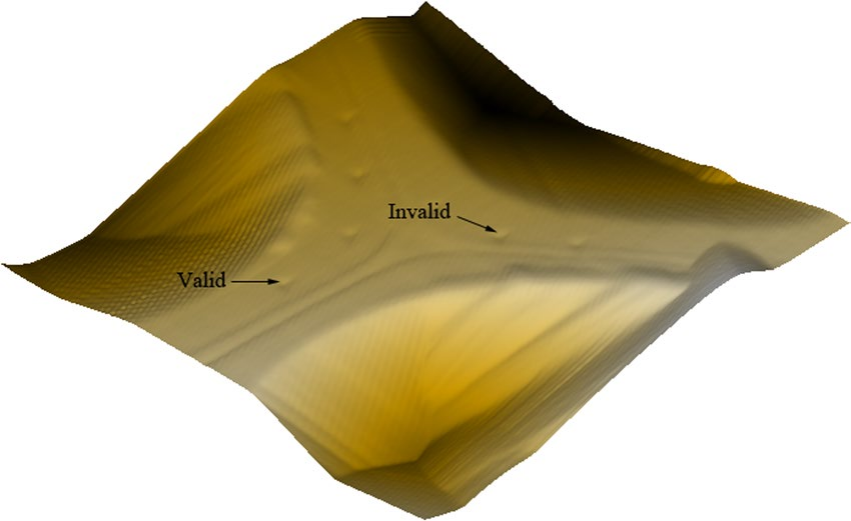
3D image of NI test on poplar cells.

**Figure 8:**
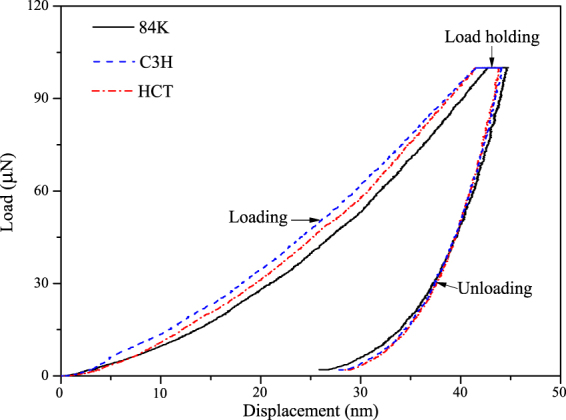
Typical NI load-displacement curves of poplar fiber cell walls.

Figure 7 shows the smooth tested surface of sample, only the indentations clearly within the secondary cell wall of a cell are valid while the invalid indentations are much closer to the compound middle lamella shared with an adjacent cell or at the edge of the cell lumen. Figure 8 shows a typical NI load-displacement curves of cell walls. MOEs and Hardnesses of samples were obtained from the valid indentations and presented in Fig. 8 and Table 8. Interestingly, MOEs of C3H and HCT transgenic poplar was 5.8% and 7.0% higher than non-modified poplar, however, the cell wall of non-modified poplar has the highest hardness values. The hardness of C3H transgenic poplar cell wall was significant lower by 10.2%. Less lignin content contributes to the lower hardness^31^. Cell wall MOEs were related to holocellulose content and cellulose crystallinity, that indicated that the increase of MOEs of both transgenic poplar might be attribute to the rise of holocellulose content and cellulose crystallinity.

**Table 8:** MOE and Hardness of non-modified poplar and transgenic poplar.

Samples	MOE (GPa)	Hardness (GPa)
Non-modified poplar	16.86 ± 0.88 A	0.528 ± 0.046 A
C3H transgenic poplar	17.84 ± 1.45 B	0.474 ± 0.036 B
HCT transgenic poplar	18.04 ± 1.20 B	0.527 ± 0.026 A
*P-value*	7.45 × 10^−3^	3.44 × 10^−4^

## Conclusion

The lignin contents in both C3H and HCT transgenic poplar wood were significantly lower than lignin conents of non-modified wood. The cellulose content were increased by 10.7% for C3H transgenic poplar compared with non-modified poplar. The hemicellulose content increased by 8.3% in HCT transgenic poplar compared with non-modified poplar. The C3H transgenic poplar and HCT transgenic poplar saplings have 18.5% and 16.1% more cellulose crystalline regions than non-modified poplar respectively.

The diameter of the fiber cells and the vessels of transgenic poplar are smaller. Double radial vessel cell wall thickness of HCT transgenic poplar is 18.5% smaller than non-modified poplar, while C3H transgenic poplar is only 3.1% smaller than non-modified poplar.

The cell wall density was significantly lower in both C3H and HCT transgenic poplar. The cell wall MOEs of C3H and HCT transgenic poplar were 5.8% and 7.0% higher than non-modified poplar respectively. The hardness of C3H transgenic poplar was 10.2% less than non-modified poplar.

HCT can be more effective than C3H to modify the trees by considerably increasing mechanical properties of the cell wall of genetic modification.

## Materials and Methods

### Plant Materials and Growth Conditions

**Figure 9:**
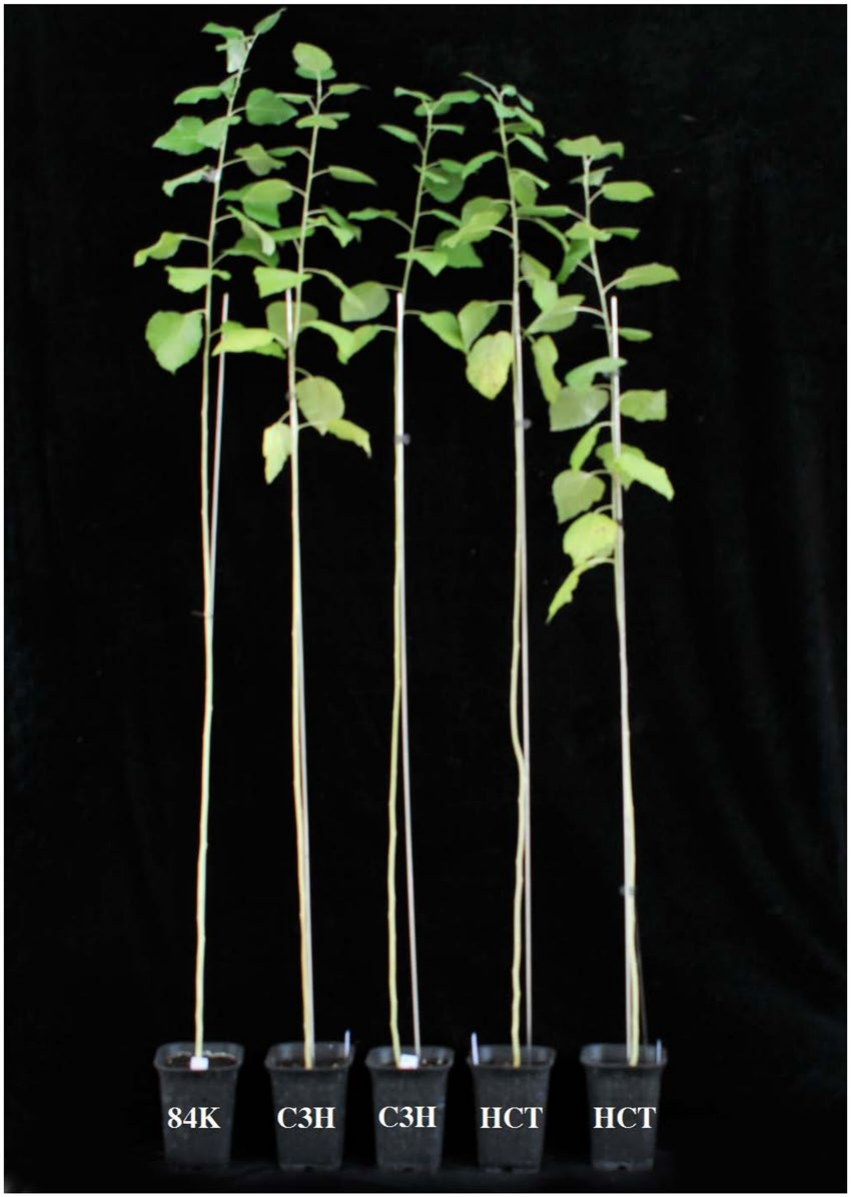
Photograph of one-year-old non-modified poplar (84 K) and HCT and C3H transgenic poplar saplings.

The transgenic poplar (*Populus alba* × *P*. *glandulosa* cv ‘84 k’) was collected as the experimental tissue and the seedlings were cultured for 1.5 months. The temperature was 22 °C with light/darkness for 16/8 hours a day. The trees were then planted in the greenhouse and watered daily. They were cultivated naturally for 6 months (Fig. 9). The expressions of C3H gene and HCT gene were 54% and 37% of non-modified plants respectively. Trees grew to an average height of 1.3 m and diameter of about 5.5 mm after 6 months. Three plants were for control. The transgenic poplar saplings were cut. And samplings were taken with the ninth internode from the tip, which was fully lignified. Internodes were taken for FTIR analysis and other measurements including NI testing, Glycosyl Linkage Assay, Anatomical explorations, Acetyl Bromide Assay, Measuring relative degree of crystallinity and Basic Density.

### Anatomical structure of generic modified poplar wood

To measure cell wall thickness and cell lumen diameter, samples were sectioned to the 15 µm thickness using a sliding microtome. The sections were dehydrated through alcohol series (30%, 5 min; 50%, 5 min; 80%, 5 min; 95%, 5 min; 100%, 10 min) and stained with safranine for 2 minutes. The cell structure was observed and studied with Leica DMLB light microscope and an image analysis system (Japan, Q570). The function of arbitrary line in the system was used to measure tangential cell lumen diameter and double radial cell wall thickness at more than 100× magnification. Three categories were chosen within annual ring from pith to bark, and 100 fibers and 100 vessel elements for each plant were measured. Cell wall ratio was measured by Image J software, and 10 categories were chosen in every sample^32^.

### Glycosyl Linkage Assay

The chemical compositions were determined with glycosyl linkage assay, 0.5 mg of 1 N KOH and 4 N KOH fractionated alcohol insoluble residues (AIRs) were methylated by incubating the residues in dimethyl sulfoxide that contains iodomethane and NaOH for 16 h^33,34^. The samples were then hydrolyzed in 2 M TFA and reduced with sodium borodeuteride in 1 M ammonium hydroxide. The partially methylated alditol acetate was analyzed by GC–MS.

### Acetyl Bromide Assay

An acetyl bromide assay was used to determine lignin content with the principle of the lignin content related to the the UV absorbance^33^. 5 mg wood flour for each sample and 5 mg alkali lignin were prepared in comparison tubes, 5 mL of freshly prepared acetyl bromide reagent, which is 30% (v/v) acetyl bromide in glacial acetic acid was added, 200 µL of perchloric acid was added to aid in the total dissolution of the wall material^35,36^, and then the tubes were capped immediately and heated in an electric-heated thermostatic water bath set at 70 °C. After heating 1 h, the samples were quantitatively transferred, with the aid of acetic acid, to 50 mL volumetric flasks that contained 10 mL of 2 M NaOH and 10 mL of acetic acid, and samples were diluted to 50 mL with acetic acid. The absorption at 280 nm for each sample was determined. All experiments were run in triplicate with duplicates for each treatment. The above alkali lignin solution was used to prepare the standard solution with concentrations of 0.01 g/L, 0.02 g/L, 0.03 g/L, 0.04 g/L and 0.05 g/L, and then draw the standard curve of alkali lignin content and UV absorbance.

### Fourier transform infrared spectroscopy (FTIR)

In FTIR testing, the epidermis, phloem and pith of saplings were removed. And then the samples were dried to air-dry and ground to wood flour. KBr flour and wood flour were mixed and oven-dried for 24 hours under the conditions of 103 ± 2 °C. Then the mixture was cooled down in a desiccator. The flour was compressed into small pellets (the diameter was 13 mm). The prepared samples were placed in an infrared light (Nicolet Impact 410, USA) to acquire the spectroscopy data, five repeats for each sample, and three samples for each transgenic poplar were analyzed. The heights of characteristic peak were obtained by OMNIC software. The ratio of characteristic peaks height of corresponding chemical composition was used to compare the content of chemical components^37–39^.

### Measuring relative degree of crystallinity (CrI%)

Three samples for each transgenic poplar sapling were measured. The flour with particle size from 0.175 to 0.246 mm was prepared by grinding the samples taken from the stems. The relative degree of crystallinity was measured with XRD. The XRD pattern was recorded within 2θ angles ranging from 5° to 40° and the scanning rate of 0.05° s^−1^. The relative degree of crystallinity (CrI%) was calculated according to equation 1 ^40^:

1 $${\rm{CrI}} \% =100({{\rm{I}}}_{002}-{{\rm{I}}}_{{\rm{am}}})/{{\rm{I}}}_{002},$$

where I_002_ represents both crystalline and amorphous parts, I_am_ represents only the amorphous part of the diffactogram.

### Basic Density

Thirty samples for each transgenic poplar sapling were used to determine their basic density, the length of them were 15 mm. The epidermis, phloem and pith were removed, sample volume was measured by the drainage method and the density was calculated.

### Nano indentation testing

Three strips in the size of 1 mm (R) × 1 mm (T) × 30 mm (L) for NI testing were prepared from the latewood. The epoxy resin was molded and they were placed in an oven at 60 °C for 36 h. The wood strips were then mounted into the embedding mold parallel to the l-axis of the cell wall using a new sample preparation method for small wood blocks without resin penetration^41^. The top surface was smoothed with a diamond knife in an ultramicrotome. The specimens were conditioned at 20 °C and a relative humidity of 65% for at least 24 h before NI test. The conditioned samples then were placed on the sample table of the nanoindenter (Triboindenter, Hysitron, USA). The diamond Berkovich indenter was used with the radius of indenter tip less than 100 nm and the taper angle of 142.35°. Quasi-static constant speed loading and unloading mode was set with the maximum load of 100 μN, the loading rate of 20 μN/s, and the loading time of 2 s. MOEs and hardnesses of samples were obtained from the valid indentations, three samplings were measured, and more than 30 valid indentations were found in every sample.

### Statistical analysis

A one-way analysis of variance (ANOVA) was used for comparative analyses. A statistical analysis of the differences among all the plants was made using a *t-test*.

### Data availability

The datasets generated during and/or analysed during the current study are available from the corresponding author on reasonable request.
